# Artificial Intelligence Control Methodologies for Shape Memory Alloy Actuators: A Systematic Review and Performance Analysis

**DOI:** 10.3390/mi16070780

**Published:** 2025-06-30

**Authors:** Stefano Rodinò, Giuseppe Rota, Matteo Chiodo, Antonio Corigliano, Carmine Maletta

**Affiliations:** Dipartimento di Ingegneria Meccanica, Energetica e Gestionale (DIMEG), University of Calabria, 87036 Rende, CS, Italy; giuseppe.rota@2smartest.com (G.R.); matteo.chiodo@2smartest.com (M.C.); antonio.corigliano@2smartest.com (A.C.); carmine.maletta@unical.it (C.M.)

**Keywords:** Shape Memory Alloys (SMAs), artificial intelligence control, smart actuators, systematic review, non-linear hysteresis compensation

## Abstract

Shape Memory Alloy (SMA) actuators are pivotal in modern engineering due to their unique thermomechanical properties, but their inherent non-linearities, hysteresis, and temperature sensitivity pose significant control challenges. This systematic review evaluates artificial intelligence (AI)-based control methodologies to address these limitations, analyzing their efficacy in enhancing precision, adaptability, and reliability for SMA and Magnetic SMA (MSMA) systems. A PRISMA-guided literature review (2003–2025) identified 24 studies, which were categorized by control architectures (hybrid AI-linear, pure AI, adaptive, and model predictive control) and evaluated through quantitative metrics, including Root Mean Square Error (RMSE%) and a weighted scoring system for experimental rigor. Results revealed hybrid AI-linear controllers as the dominant approach (36%), with online-trained neural networks achieving superior accuracy (+2.4%) over offline methods. Feedforward neural networks outperformed recurrent architectures (+3.1%), while Model Predictive Control (MPC) excelled for SMA actuators (+5.8% accuracy) but underperformed for MSMAs (−7.7%). Sensorless strategies proved advantageous for MSMAs (+5.0%), leveraging intrinsic material properties like electrical resistance for state estimation. The analysis underscores AI’s capacity to mitigate hysteresis and non-linear dynamics, though material-specific optimization is critical: SMA systems favor dynamic control and MPC, whereas MSMAs benefit from sensorless AI and pure neural networks. Challenges persist in computational demands for online training and reinforcement learning’s exploration–exploitation trade-offs. Future research should prioritize adaptive algorithms for fatigue compensation, lightweight AI models for embedded deployment, and standardized benchmarking to bridge material-specific performance gaps. This synthesis establishes AI as a transformative paradigm for SMA actuation, enabling precise control in aerospace, biomedical, and soft robotics applications.

## 1. Introduction

Shape Memory Alloys (SMAs) represent a class of smart materials that have garnered significant attention in engineering applications due to their unique ability to “remember” and recover their original shape when subjected to specific thermal or mechanical stimuli [1]. This remarkable property, known as the shape memory effect, enables SMAs to function as both sensors and actuators simultaneously, making them particularly valuable in applications where conventional actuators face limitations due to space constraints, weight requirements, or complex mechanical designs [2,3]. SMA actuators have emerged as a compelling alternative to traditional actuators in various engineering domains. Their ability to generate substantial forces over considerable displacements while maintaining a relatively low weight profile makes them increasingly sought after in applications ranging from aerospace and automotive industries to biomedical devices and robotics [4,5,6,7]. The high power-to-weight ratio of SMA actuators, combined with their silent operation and mechanical simplicity, positions them as ideal candidates for specialized applications where conventional electromagnetic or hydraulic actuators would be impractical.

In the field of rehabilitation robotics, for instance, SMA actuators offer the potential for creating lightweight, compliant devices that can safely interact with human users [8,9]. Their inherent compliance and biomimetic behavior resembling natural muscle function make them particularly suitable for wearable assistive devices and prosthetics [10]. Similarly, in aerospace applications, the weight reduction enabled by SMA actuators can translate to significant fuel savings and enhanced payload capacity [11,12].

The growing interest in soft robotics has further accelerated research into SMA actuators, as these materials can be integrated into flexible structures to create bio-inspired robotic systems capable of complex movements and adaptations to unstructured environments [13,14]. This versatility extends to microelectromechanical systems (MEMS), where SMA actuators can provide reliable actuation at microscales with minimal complexity [15]. Recent advancements in SMA actuator design have addressed the traditional limitation of slow actuation speed. Song et al. [16] demonstrated a smart soft composite actuator achieving 35 Hz operation through the use of multiple thin SMA wires to enhance heat dissipation, with resonance effects enabling large deformations. This approach represents a significant leap from conventional SMA actuators typically limited to a few Hz. Furthermore, thin-film SMA technologies have enabled kHz-frequency operation in MEMS applications, as demonstrated by Stachiv et al. [17] in tunable high-frequency microcantilever resonators using NiTi films. These developments highlight the expanding operational envelope of SMA actuators and the increasing importance of precise control methodologies.

Despite their promising attributes, the widespread adoption of SMA actuators has been hindered by significant challenges in their control. The behavior of SMAs is characterized by pronounced non-linearities and hysteresis, which complicate the development of precise and reliable control systems [1,18,19]. These inherent characteristics stem from the complex thermomechanical processes that govern the phase transformations within the material. The primary challenges in SMA control include the following:

1. Non-linear Behavior: The relationship between temperature, stress, and strain in SMAs is highly non-linear, making it difficult to predict and control their response using conventional linear control techniques.
2. Hysteresis: SMAs exhibit significant hysteresis, meaning that their current state depends not only on the present input but also on their previous states. This path-dependent behavior creates a memory effect that complicates control strategies.
3. Temperature Dependence: The actuation of SMAs is fundamentally temperature-driven, making their performance sensitive to ambient temperature variations and thermal management challenges [20].
4. Rate Dependence: The response rate of SMAs is limited by heating and cooling dynamics, resulting in relatively slow actuation speeds compared to conventional actuators [19].
5. Fatigue and Degradation: Over repeated cycles, SMAs can experience performance degradation, affecting the consistency and reliability of their response [21,22].

These challenges have motivated extensive research into advanced control methodologies capable of addressing the complex behavior of SMA actuators. While conventional control approaches have shown limited success in handling these complexities, artificial intelligence (AI) methods have emerged as promising alternatives due to their ability to model and adapt to non-linear and hysteretic systems [18,19]. A fundamental premise underlying this systematic review is the equivalent treatment of conventional Shape Memory Alloys (SMAs) and Magnetic Shape Memory Alloys (MSMAs) within our analytical framework. This approach is scientifically justified by the observation that both material systems exhibit identical core control challenges: pronounced hysteresis behavior, complex non-linear thermomechanical coupling, temperature-dependent response characteristics, and phase transformation dynamics. The distinction between thermal actuation (conventional SMAs) and magnetic field actuation (MSMAs) represents a difference in input modality rather than fundamental control behavior, making unified analytical treatment both appropriate and necessary for comprehensive systematic analysis. This unified approach enables robust quantitative analysis across a broader dataset while accurately reflecting the transferability of AI control methodologies between material variants. The natural progression of the field, wherein foundational control understanding developed through conventional SMA research subsequently transferred to MSMA applications, supports this methodological decision and strengthens the generalizability of our findings across smart material actuation systems.

The paper is organized as follows. Section 2 presents a detailed overview of Shape Memory Alloy (SMA) actuators, including their fundamental principles, actuation mechanisms, and bibliometric insights derived from a systematic literature review. Section 2 presents a detailed overview of Shape Memory Alloy (SMA) actuators, including their fundamental principles, actuation mechanisms, and bibliometric insights derived from a systematic literature review. Section 3 critically examines conventional and artificial intelligence-based control methodologies for SMA actuators, focusing on architectural classifications, feedback strategies, and control performance metrics. This section is subdivided into three main components:

- Section 3.1 analyzes sensing technologies and instrumentation approaches for SMA actuator systems;
- Section 3.2 provides a comprehensive evaluation of classical control strategies, including their architectural classifications, implementation characteristics, and performance analysis;
- Section 3.3 delivers an in-depth assessment of artificial intelligence techniques, encompassing feedforward neural networks, recurrent architectures, LSTM networks, and reinforcement learning approaches employed in SMA control, with particular emphasis on their training modalities, adaptability, and implementation efficacy.

The analysis concludes with a systematic evaluation of AI control limitations and establishes a comprehensive research roadmap for future developments in intelligent SMA actuation systems, identifying prevailing research gaps and proposing strategic directions for advancing the field of AI-controlled smart actuators.

## 2. SMA Actuators and Literature Review

### 2.1. Literature Review: Methodology and Bibliometric Data

This review paper aims to provide a comprehensive analysis of artificial intelligence control methods for SMA actuators, with particular emphasis on their effectiveness in addressing the inherent challenges of SMA control. The specific objectives of this review include the following:

1. Examining the fundamental principles and characteristics of SMAs that influence their control requirements;
2. Analyzing conventional control approaches and their limitations when applied to SMA actuators;
3. Investigating various AI-based control methodologies and their applications to SMA systems;
4. Evaluating the performance of different AI control strategies through comparative analysis;
5. Identifying current challenges, emerging trends, and future research directions in AI-controlled SMA actuators.

**Figure 1:**
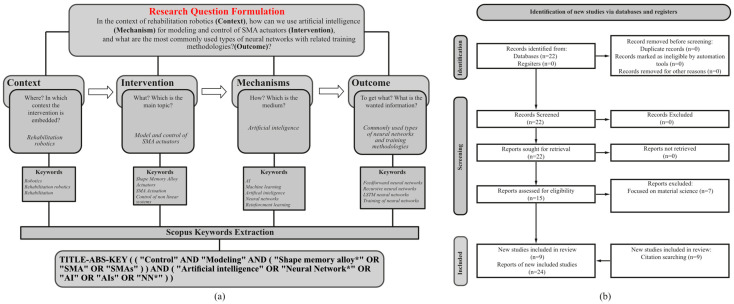
(**a**) CIMO logic scheme for formulating the research question. (**b**) PRISMA flowchart showing the literature screening process.

To achieve these objectives, a comprehensive systematic review methodology was implemented following the PRISMA 2020 (Preferred Reporting Items for Systematic Reviews and Meta-Analyses) framework. This research adhered to all 27 items specified in the PRISMA 2020 checklist to ensure methodological rigor and transparency in the systematic identification, selection, and analysis of relevant literature [23]. The complete PRISMA 2020 checklist has been fulfilled, and a corresponding flow diagram documenting the study selection process is presented in Figure 1b.

The review process began with the formulation of a research question using the CIMO (Context, Intervention, Mechanism, Outcome) framework as reported in Figure 1a.

This question guided the development of a comprehensive search strategy implemented in the Scopus database, initially focusing on publications from 2015 to 2025 in the English language, within engineering and computer science domains. The initial search yielded 22 articles, which were subsequently screened based on their abstracts to eliminate off-topic papers (primarily those focused on material characterization rather than control). This screening process reduced the pool to 15 relevant articles. Through citation analysis of these papers, an additional 13 articles were identified that were either not indexed in Scopus or not captured by the selected keywords, bringing the total to 28 articles.

After a comprehensive review of the full texts, four articles focusing solely on SMA modeling without control algorithms were excluded, resulting in a final selection of 24 articles for detailed analysis. The review also considered papers on Magnetic Shape Memory Alloys (MSMAs) due to their similar non-linear and hysteretic behavior, which presents comparable control challenges to conventional SMAs. All the relevant steps of the research are well described in logic flow of Figure 1b.

**Figure 2:**
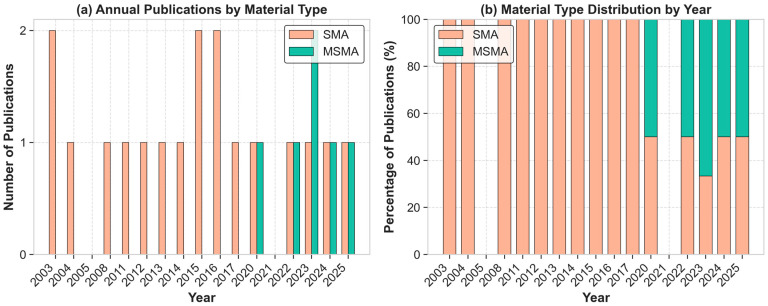
Temporal distribution of SMA and MSMA research publications (2003−2025).

**Figure 3:**
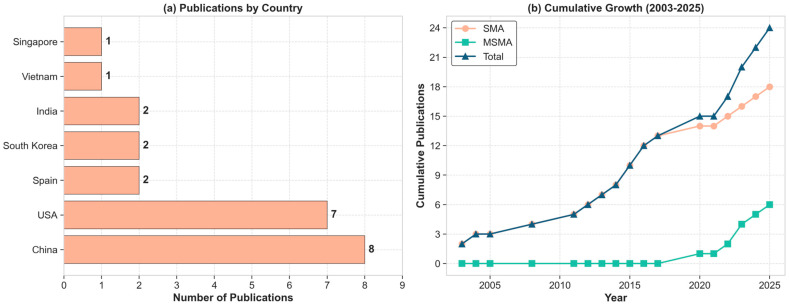
Cumulative growth of SMA and MSMA research publications.

The final selected articles span from 2003 to 2025, with a notable shift in focus from conventional SMAs (predominant from 2003 to 2021) to MSMAs in more recent years as reported in Figure 2 and Figure 3.

**Figure 4:**
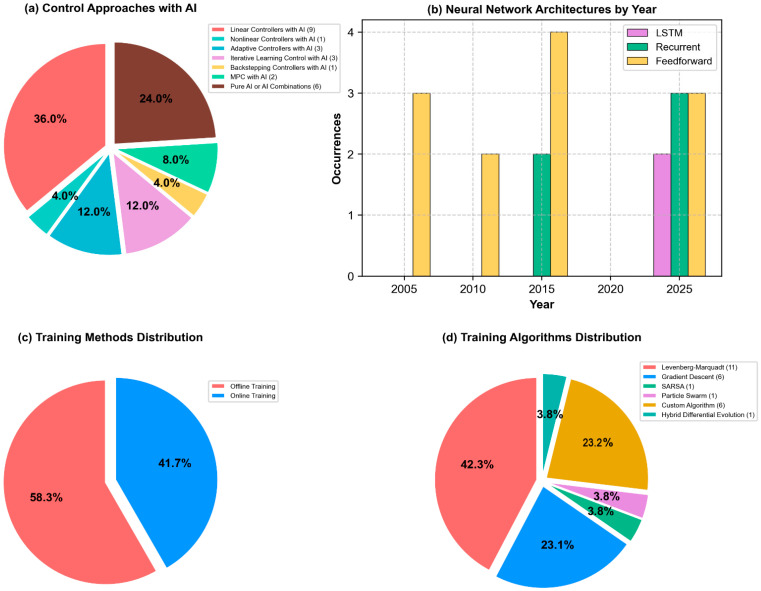
AI methods of SMA actuators control in the literature (2003−2025).

Figure 4 presents a comprehensive classification of control methodologies identified in the systematic review of SMA and MSMA actuator literature from 2003 to 2025. Figure 4a demonstrates the predominance of hybrid approaches combining linear controllers with AI techniques (36%), followed by pure AI-based systems (24%). Less prevalent methodologies include adaptive controllers, Iterative Learning Control, backstepping control, Model Predictive Control and Non-linear Control Systems with AI components. Figure 4b shows feedforward architectures which represent the dominant strategy. Figure 4c reveals that 58.3% of all implementations utilize neural network trained offline. Finally, Figure 4d illustrates the distribution of AI training methodologies. Recent publications suggest a gradual shift toward online methodologies, reflecting advancements in computational capabilities and increased recognition of adaptation requirements for SMA and MSMA control.

**Figure 5:**
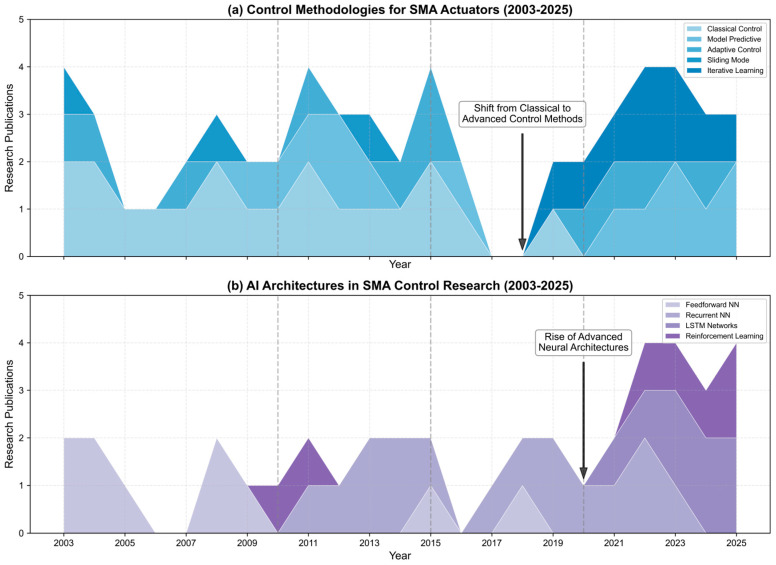
Temporal evolution of research priorities in SMA actuator control from 2003 to 2025: (**a**) The streamgraphs illustrate the shifting focus across control methodologies and (**b**) AI architectures (bottom).

Figure 5 illustrates the evolution of research focus in SMA actuator control from 2003 to 2025. The visualization reveals a clear transition from classical control methodologies toward advanced AI-based approaches. Notably, while feedforward neural networks dominated early AI implementations (2003–2010), recent years show significant growth in recurrent architectures and LSTM networks (post-2020).

### 2.2. Shape Memory Alloy Fundamentals

Shape Memory Alloys (SMAs) represent a unique class of metallic materials that exhibit remarkable thermomechanical properties, distinguishing them from conventional engineering metals and alloys. The exceptional properties of SMAs originate from a reversible, diffusion less solid-state phase transformation between two crystalline structures: austenite and martensite [1]. Austenite, the high-temperature phase, exhibits a cubic crystal structure that is relatively symmetric and stable at elevated temperatures. Martensite, the low-temperature phase, possesses a more complex, less symmetric crystal structure that can exist in multiple variants [24].

**Figure 6:**
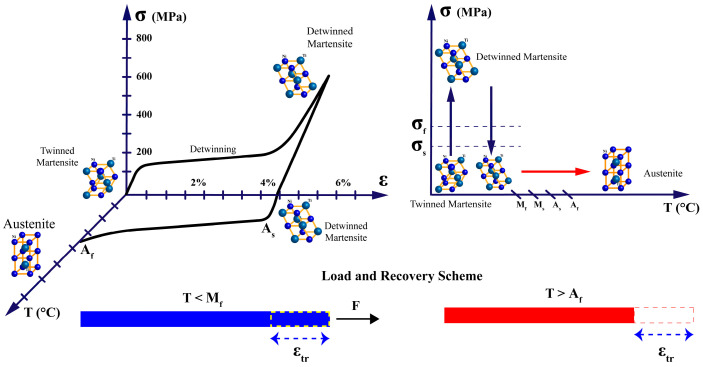
One-way shape memory effect (SME) in SMA actuators.

**Figure 7:**
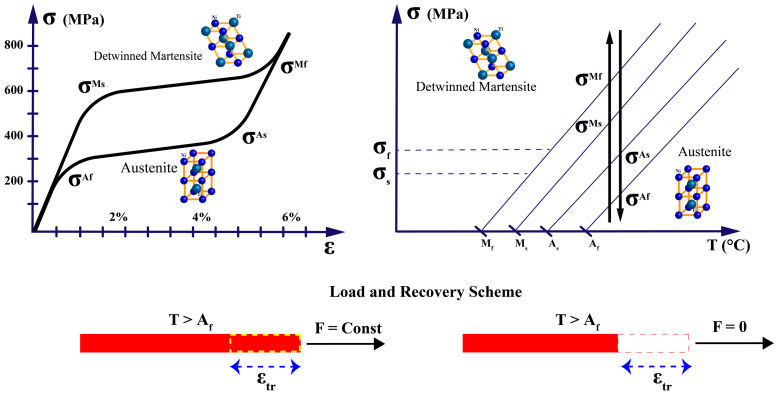
Superelasticity (pseudoelasticity) in SMA actuators.

The transformation between these phases can be induced by changes in temperature or applied stress, and it is this transformation that underpins the two primary phenomena observed in SMAs:

1. **Shape Memory Effect (SME)**: When a SMA in its martensitic phase is deformed and subsequently heated above its transformation temperature, it recovers its original shape as it transforms to austenite. Upon cooling, the material returns to martensite without macroscopic shape change unless external stress is applied, as in Figure 6. This one-way shape memory effect is the basis for most SMA actuator applications.
2. **Superelasticity (or Pseudoelasticity)**: When an SMA is at a temperature above its austenitic finish temperature, the application of mechanical stress can induce a transformation to stress-induced martensite. Upon removal of the stress, the material spontaneously reverts to austenite, recovering its original shape, as in Figure 7. This property allows SMAs to undergo large deformations (up to 8–10% strain) and return to their original shape without permanent deformation.

The phase transformation in SMAs is characterized by four critical temperatures:

- **Martensite start (M_s_)**: Temperature at which martensite begins to form during cooling.
- **Martensite finish (M_f_)**: Temperature at which the transformation to martensite completes during cooling.
- **Austenite start (A_s_)**: Temperature at which austenite begins to form during heating.
- **Austenite finish (A_f_)**: Temperature at which the transformation to austenite completes during heating.

**Figure 8:**
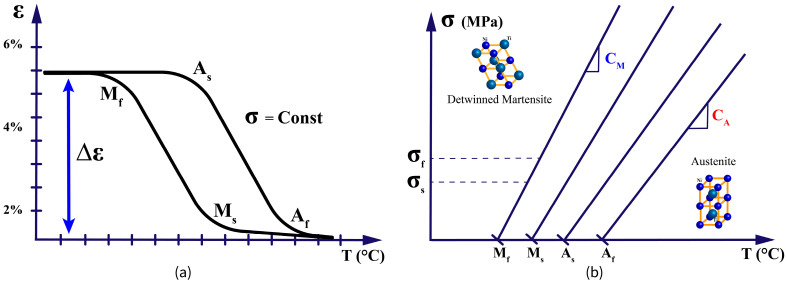
(**a**) Variability of transformation temperatures (TTs) under applied stress and (**b**) Typical phase transformation behavior during thermal cycling.

These transformation temperatures (TTs) are not fixed material constants but depend on factors such as alloy composition, processing history, and applied stress [25]. The relationship between these temperatures and the phase transformation is illustrated in Figure 8a,b, which shows the variability of the TTs against the applied stress and the typical transformation behavior of during thermal cycling.

The thermomechanical behavior of SMAs is characterized by a complex interplay between temperature, stress, and strain. Understanding these relationships is crucial for designing effective control strategies for SMA actuators. The mechanical response of SMAs is highly temperature-dependent, with distinct behavior observed in different temperature regimes:

1. **Below M_f_**: The material is fully martensitic and exhibits a detwinning plateau in its stress–strain curve, allowing for large recoverable deformations through the reorientation of martensite variants.
2. **Between M_f_ and A_f_**: The material exists in a mixed-phase state, with the proportion of austenite increasing with temperature. The mechanical properties in this region are highly sensitive to temperature changes.
3. **Above A_f_**: The material is fully austenitic and exhibits superelastic behavior, with stress-induced martensite forming under load and reverting to austenite upon unloading.

The stress–strain curves of SMAs also exhibit significant temperature dependence. As temperature increases, the critical stress required for martensitic transformation increases linearly according to the Clausius–Clapeyron relationship [26]:

(1) $$\frac{d\sigma }{dT}=-\frac{\Delta H}{\varepsilon_{0}T_{0}},$$

where σ is the critical stress, *T* is the temperature, ΔH is the latent heat of transformation, $\varepsilon_{0}$ is the transformation strain, and *T*_0_ is the equilibrium temperature.

### 2.3. Types of SMA Actuators

**Figure 9:**
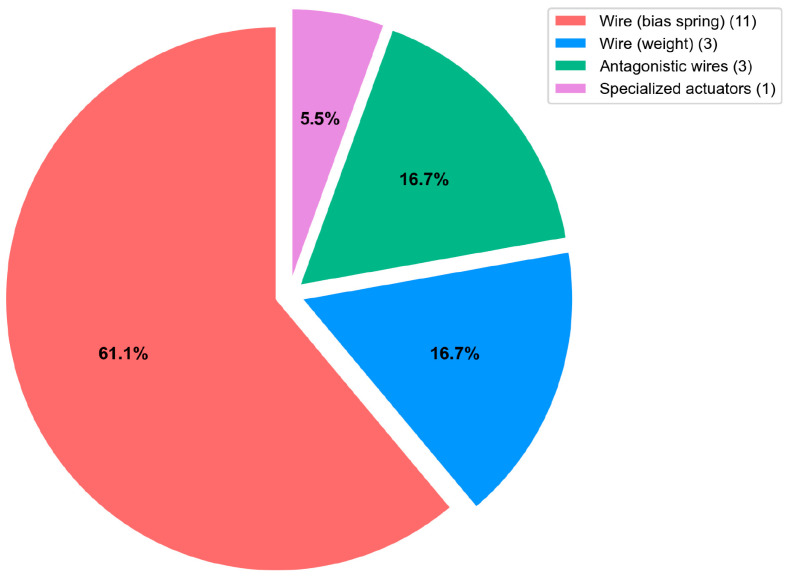
Classification and distribution of SMA actuator types by mechanical configuration.

Shape Memory Alloy (SMA) actuators can be classified by their mechanical configuration, which determines their performance characteristics across parameters including displacement amplitude, force generation, actuation frequency, and energy efficiency. The four categories (Wire (bias spring), Wire (weight), Antagonistic wires, Specialized actuators) analyzed in this work and relative distribution are reported in Figure 9.

**Figure 10:**
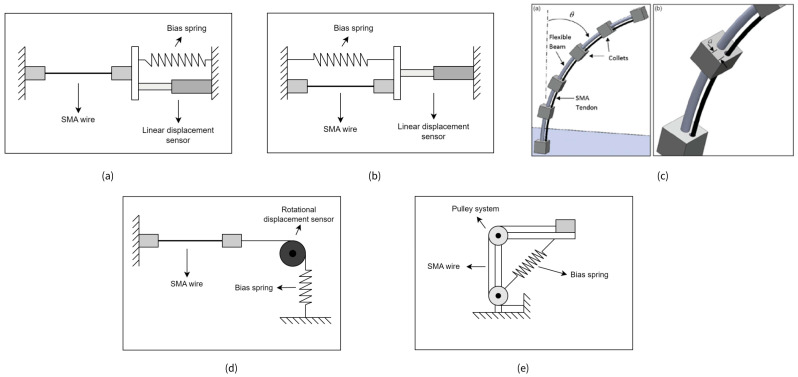
SMA actuator configurations with bias mechanisms. (**a**) Axial motion system with serial arrangement [27]; (**b**) experimental setup with parallel configuration [28]; (**c**) flexible beam with SMA “tendon” generating bending moment [29]; (**d**) rotary motion system with serial spring arrangement [30]; (**e**) pulley-based system with parallel spring configuration [31].

**Figure 11:**
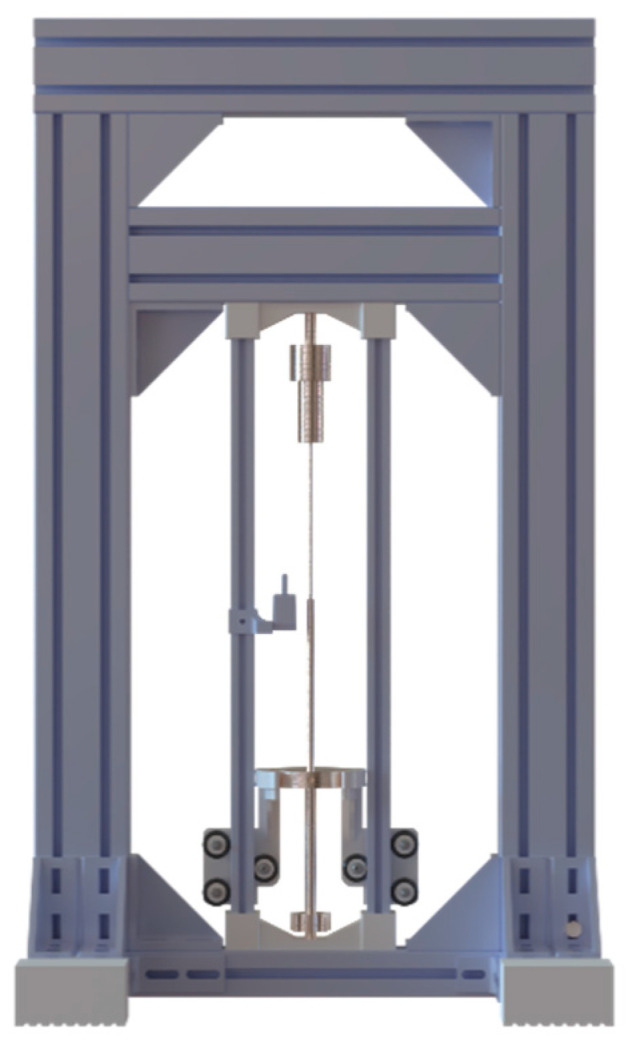
SMA actuator configurations with hanging mass as a resetting mechanism.

**Figure 12:**
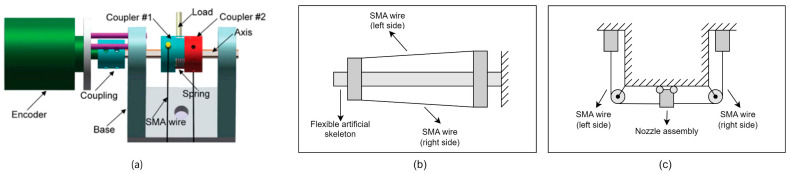
Antagonistic SMA wire configurations. (**a**) Experimental setup mimicking joint articulation with antagonistic SMA wires [32]; (**b**) biomimetic fish tail system using antagonistic SMA wires for bidirectional actuation [33]; (**c**) precision positioning mechanism with antagonistic SMA wire arrangement for accurate nozzle movement in FDM 3D printing [34].

**Figure 13:**
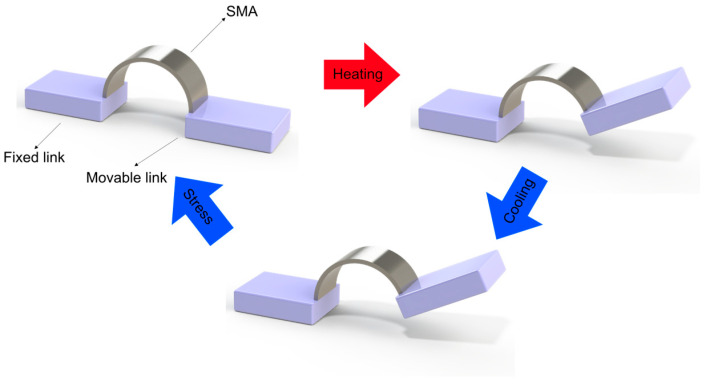
Specialized arc-shaped SMA actuator generating rotational motion [35].

- **Wire actuators with bias spring** represent the most prevalent configuration (11 documented cases), utilizing a mechanical spring to provide the restoring force necessary for shape recovery during cooling. When heated, the SMA wire contracts against the bias spring; upon cooling, the spring extends the wire to its original length. This configuration offers mechanical simplicity with predictable force-displacement relationships, though it introduces parasitic energy consumption as the SMA must work against spring resistance. Multiple geometric arrangements are possible:
  ○ Serial or parallel configurations for axial motion generation, Figure 10a,b;
  ○ Beam-mounted “tendon” arrangements generating bending moments, Figure 10c;
  ○ Pulley systems with torsional springs for rotary motion, Figure 10d,e.

- **Wire actuators with weight** (three documented cases) employ gravitational loading as the resetting mechanism. This configuration improves thermomechanical efficiency by eliminating energy losses associated with working against variable spring resistance. However, it requires specific orientation considerations and offers limited implementation flexibility. These configurations are commonly employed in experimental setups due to their constant-force characteristics throughout the displacement range, Figure 11.

- **Antagonistic wire configurations** (three documented cases) utilize opposing SMA elements for bidirectional actuation without passive resetting mechanisms. Primary advantages include active bidirectional control capabilities and enhanced positioning precision, though with increased control complexity. Applications include the following:
  ○ Soft robotic systems mimicking biological joint articulation, Figure 12a;
  ○ Biomimetic structures such as fish-tail locomotion mechanisms, Figure 12b;
  ○ Precision positioning systems for manipulation tasks, Figure 12c.

- **Specialized actuator designs** (one documented case) encompass various geometries addressing specific performance requirements. The documented example features an arc-shaped SMA element generating rotational motion without additional mechanical components (Figure 13) [35], demonstrating the adaptability of control methodologies across diverse geometrical configurations.

## 3. Conventional Control Methods for SMA Actuators

### 3.1. Sensing Technologies for SMA Actuators

Experimental setups for Shape Memory Alloy (SMA) actuators require sophisticated sensing technologies to monitor various parameters during operation. These sensors serve critical functions in both the development and implementation phases, providing essential feedback for control system calibration, performance verification, and real-time operation. This section examines the diverse sensing technologies employed in SMA actuation systems, categorized by their measurement objectives and implementation methodologies.

#### 3.1.1. Position Sensors

**Figure 14:**
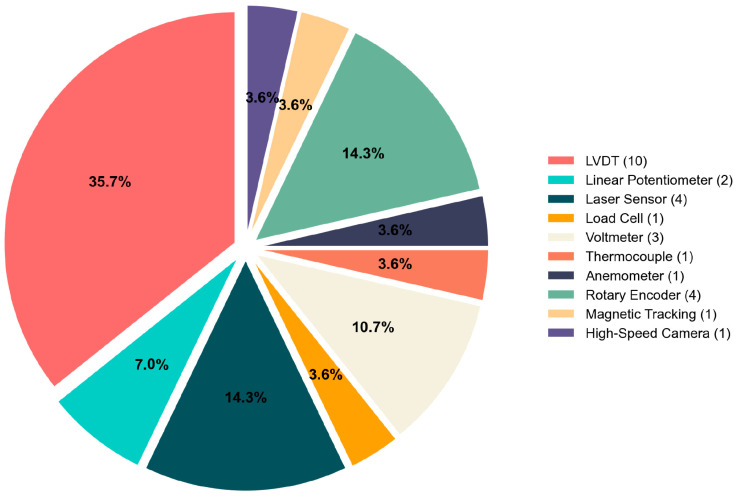
Position sensing technologies for SMA actuators, categorized by measurement principles and applications.

Position sensing represents the most fundamental measurement requirement for SMA actuator systems, with position sensors present in all experimental setups examined in the literature. These sensors provide critical displacement data for controller calibration, feedback signals, and tracking error assessment [36,37,38]. The selection of appropriate position sensors depends primarily on the actuator geometry and the motion characteristics being monitored, with several predominant technologies emerging across research implementations, Figure 14.

The systematic categorization of position sensing technologies for SMA actuators reveals a diverse instrumentation landscape, each offering distinct operational capabilities suited to specific experimental requirements. Table 1 presents a comprehensive analysis of these sensing modalities, delineating their measurement principles, precision characteristics, and application domains.

#### 3.1.2. Alternative Sensing Methodologies

The comprehensive characterization and control of SMA actuator systems necessitates sensing capabilities beyond conventional position monitoring. Table 2 presents a systematic classification of alternative sensing methodologies that leverage both intrinsic material properties and external measurement approaches to provide complementary data streams for enhanced system understanding and control implementation.

The systematic implementation of these complementary sensing approaches, in conjunction with the position measurement technologies discussed previously, establishes a comprehensive instrumentation framework for advanced SMA actuator research and development, balancing measurement precision requirements against system integration constraints for optimized performance characterization and control implementation.

### 3.2. Classical Control Strategies

The complex non-linear behavior and hysteretic characteristics of Shape Memory Alloy (SMA) actuators necessitate sophisticated control methodologies to achieve precise, reliable performance. Analysis of the contemporary research landscape reveals distinct approaches to SMA control, which can be categorized according to several defining parameters, including adaptation capability, feedback methodology, and sensor utilization.

#### 3.2.1. Control Architecture Classification

**Figure 15:**
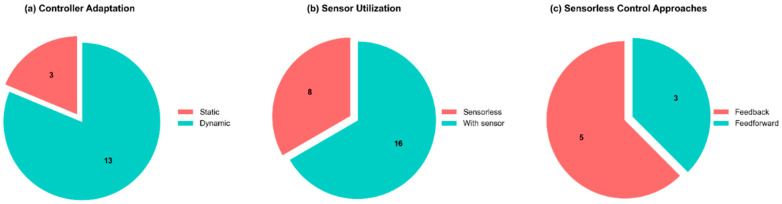
Classification of SMA control strategies: (**a**) static vs. dynamic control implementations, (**b**) sensor vs. sensorless utilization, and (**c**) open-loop vs. closed-loop architectures.

The primary classification of SMA control strategies emerges from their fundamental architecture, which determines both implementation complexity and operational capabilities. Figure 15 presents a systematic categorization of the control approaches identified in the literature.

- **Static vs. Dynamic Control:** The temporal adaptation characteristics of control systems represent another critical distinction, with static controllers maintaining fixed parameters throughout operation and dynamic controllers implementing parameter adjustment mechanisms to accommodate system evolution over time. The literature exhibits a strong preference for dynamic controllers (13 of 16 closed-loop implementations) as reported in Figure 15a, which continuously adjust control parameters to adapt to the evolving hysteretic characteristics that develop during cyclic actuation [47]. All adaptive implementations rely on position sensor feedback for parameter updating, with no sensorless adaptive solutions identified in the current research landscape.
- **Sensor Utilization**: In more than half of the examined studies (16 of 24), position sensors provide essential feedback during controller operation, enabling real-time state monitoring and adjustment (Figure 15b). The remaining implementations (eight studies) utilize sensorless approaches that eliminate position sensors during operation, though these sensors remain present during the experimental characterization and control system calibration phases. Sensorless implementations offer significant advantages for miniaturized applications by eliminating bulky position sensors that reduce system flexibility and constrain integration potential.
- **Open-Loop vs. Closed-Loop Control**: The research corpus demonstrates a clear predominance of closed-loop control architectures, with only 8 of the 24 investigated studies implementing open-loop strategies. This distribution reflects the inherent limitations of open-loop approaches, which apply predefined actuation signals without compensating for system state variations or external disturbances, thereby providing limited effectiveness in addressing the non-linearities and hysteresis inherent to SMA actuators [28,38].
- **Feedforward control**: open-loop controllers employed in SMA systems typically implement feedforward architectures that incorporate predictive compensation for system uncertainties, particularly hysteresis phenomena. A notable subset of these controllers (five of eight open-loop implementations) integrate estimator modules, predominantly neural network structures, to evaluate actuator displacement without physical sensors, thereby creating virtual feedback loops that enable closed-loop operational characteristics while maintaining the implementation advantages of sensorless operation (Figure 15c).

Within these architectural frameworks, various control algorithms have been implemented for SMA actuation, with notable concentration in several methodological categories:

1. **Linear Controllers with AI Integration:** The most prevalent approach combines classical linear controllers with artificial intelligence algorithms, leveraging the stability characteristics of conventional control while addressing non-linearities through AI-based compensation mechanisms.
2. **Pure AI Controllers:** Control systems based exclusively on artificial intelligence methodologies represent the second most common implementation strategy, employing various neural network architectures to capture the complex behavior of SMA systems.
3. **Non-Linear Control with AI Enhancement:** Several studies implement non-linear control techniques such as Sliding Mode Control (SMC) in conjunction with AI algorithms, capitalizing on the robustness of SMC approaches while mitigating their limitations through intelligent adaptation.
4. **Specialized Control Methodologies:** Less common implementations include adaptive controllers, Iterative Learning Control (ILC), backstepping controllers, and Model Predictive Control (MPC), each offering specific advantages for particular operational requirements.

The subsequent sections will examine these methodological approaches in detail, analyzing their implementation architectures, operational characteristics, and performance metrics to establish a comprehensive assessment of their relative efficacy for SMA actuator control.

#### 3.2.2. Type of Control Strategies

This section presents a systematic comparison of fundamental control strategies that have demonstrated efficacy in SMA/MSMA actuation systems. The comparative framework encompasses six principal methodologies: Proportional-Integral-Derivative (PID) control, Sliding Mode Control (SMC), Backstepping Dynamic Surface Control (BDSC), Adaptive Control, Model Predictive Control (MPC), and Iterative Learning Control (ILC). Each approach offers distinct advantages and limitations when applied to the unique thermomechanical characteristics of shape memory materials.

Table 3 provides a structured analysis of these control strategies, examining their classification, working principles, key advantages, implementation challenges, and specific applicability to SMA/MSMA actuation systems.

#### 3.2.3. Control Strategy Performance Analysis

The systematic evaluation of performance characteristics across diverse control methodologies for Shape Memory Alloy (SMA) and Magnetic Shape Memory Alloy (MSMA) actuators necessitates a standardized quantitative framework capable of facilitating meaningful comparative assessment. This section delineates the analytical methodology employed for performance quantification and introduces the comprehensive comparative matrix synthesizing findings across the literature.

The analytical evaluation of control strategy efficacy employs Root Mean Square Error (RMSE) as the primary performance metric, providing a mathematically rigorous measure of tracking precision. This statistical parameter offers a comprehensive representation of error dispersion around the reference trajectory through quadratic processing of deviation magnitudes. The mathematical formulation of RMSE is expressed as follows:

(2) $$\mathrm{RMSE}=\sqrt{\frac{\sum {\left(y_{\mathrm{Desired}}-y_{\mathrm{Obtained}}\right)}^{2}}{N}}$$

where $y_{\mathrm{Desired}}$ represents the target position trajectory, $y_{\mathrm{Obtained}}$ denotes the actual position achieved by the actuator, and N signifies the number of sampling points within the evaluation interval.

To facilitate comparative analysis across actuator systems with heterogeneous displacement ranges, normalization of RMSE values with respect to the total actuator stroke (Δy) yields a dimensionless percentage metric (RMSE%):

(3) $$RMSE\%=\frac{RMSE}{\Delta y}100$$

The comparative evaluation of control methodologies for Shape Memory Alloy (SMA) and Magnetic Shape Memory Alloy (MSMA) actuators presents significant analytical challenges due to heterogeneous testing protocols and performance metrics implemented across independent investigations. To address these methodological limitations and establish a standardized comparative framework, we have developed a weighted scoring system that integrates both tracking precision and experimental comprehensiveness through a systematic multi-parameter assessment protocol.

The scoring methodology quantifies experimental rigor through binary classification (presence = 1, absence = 0) across six critical experimental parameters:

- **Periodic Signal Testing**: Implemented as a baseline evaluation metric present across all studies;
- **Signal Typology Diversity**: Presence of multiple reference trajectories with distinct morphological characteristics;
- **Amplitude Variation**: Implementation of variable amplitude parameters within testing protocols;
- **Frequency Modulation**: Incorporation of frequency variability within reference trajectories;
- **Load Variation**: Post-calibration performance assessment under diverse loading conditions;
- **External Disturbance Introduction**: Explicit incorporation of input/output perturbations.

For each controller implementation, these parameters are aggregated to form a composite weighting factor ($P_{\mathrm{Controller}}$), as defined by Equation (4):

(4) $$P_{\mathrm{Controller}}=\sum {\mathrm{Weights}}_{\mathrm{Controller}}$$

The composite weighting factor is subsequently multiplied by the demonstrated accuracy (1−RMSE%) to derive a “weighted accuracy score” according to Equation (5):

(5) $$Weighted\_Accuracy_{\mathrm{Controller}}=P_{\mathrm{Controller}}\left(1-\mathrm{RMSE}\%\right)$$

The quantitative performance metrics (RMSE% based) derived from various test protocols are systematically presented in Table 4, alongside methodological classifications for each control strategy. For multi-condition studies, maximum error values are reported with appropriate annotation. The table incorporates a derived “accuracy score” metric that integrates experimental complexity with error quantification, with calculation methodology detailed in subsequent analysis.

**Figure 16:**
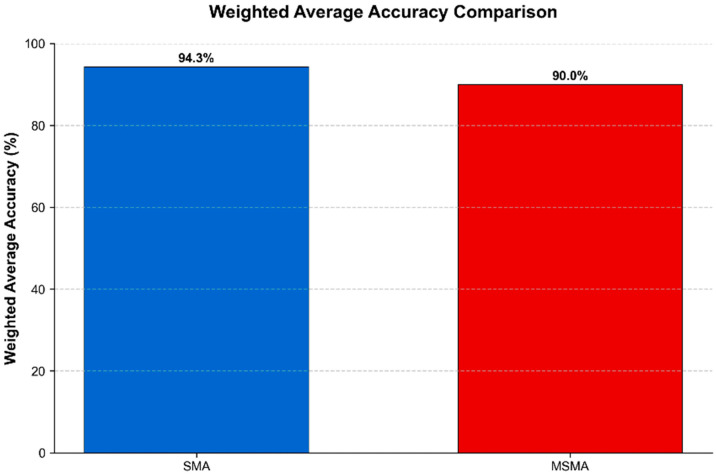
Comparative analysis of weighted average accuracy between SMA and MSMA actuator control systems.

Statistical aggregation of performance metrics across actuator categories revealed significant performance differentials between SMA and MSMA control implementations. Controllers designed for conventional SMA actuators demonstrated superior tracking precision (weighted average accuracy: 0.943) compared to their MSMA counterparts (weighted average accuracy: 0.900), Figure 16. This performance differential is attributed to the heightened complexity of magneto-mechanical coupling phenomena and increased sensitivity to external magnetic field variations inherent to MSMA systems.

**Figure 17:**
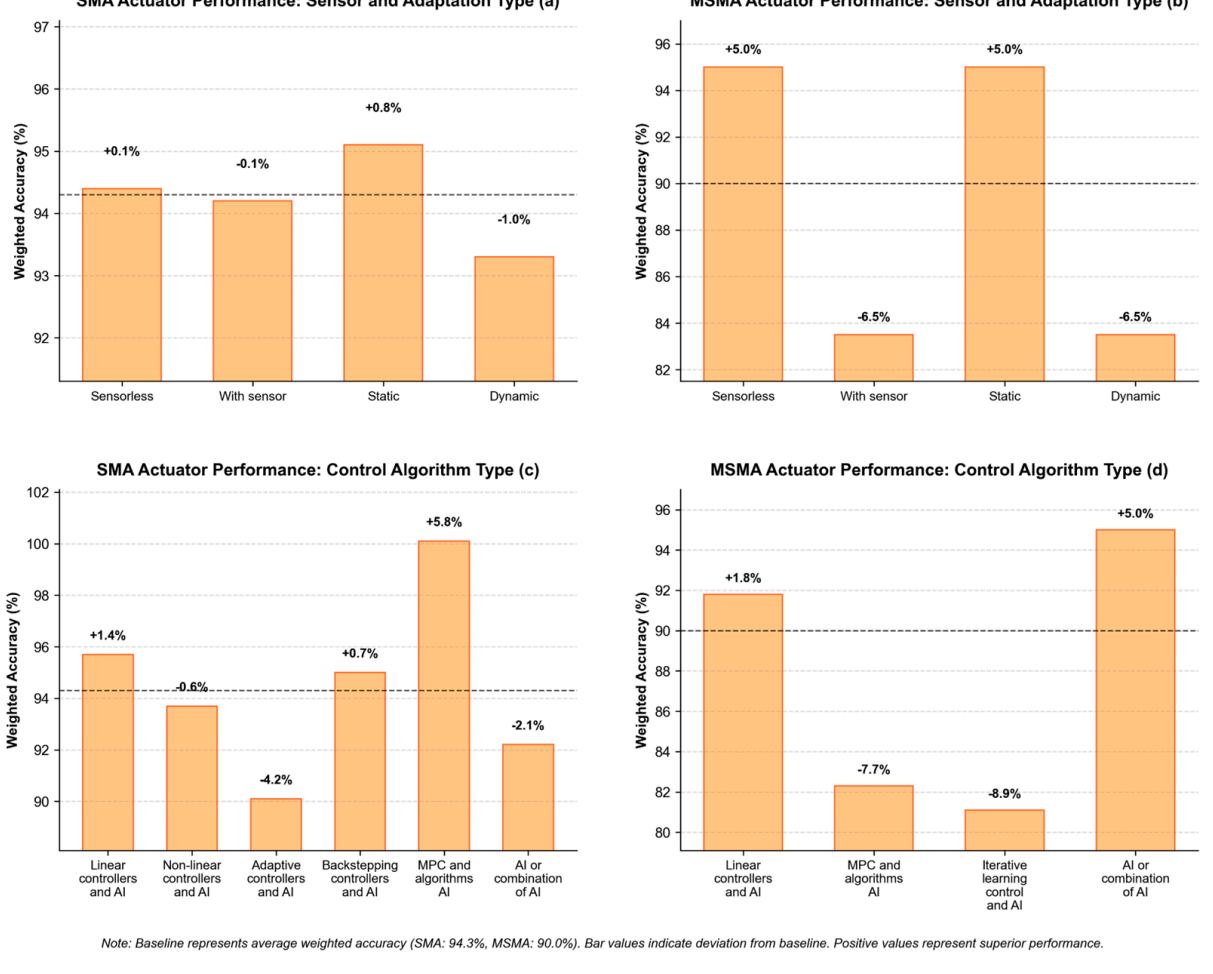
Comparative analysis of control strategies for SMA and MSMA actua-tors, where dashed line represents the average Weighted Accuracy (%).

The multi-panel visualization of Figure 17 presents performance differentials across control implementations for Shape Memory Alloy (SMA) and Magnetic Shape Memory Alloy (MSMA) actuators. Quantitative analysis reveals distinct optimization pathways for each material system.

SMA actuators demonstrate superior performance under static control conditions (+0.8%) compared to dynamic implementations (−1.0%), with Model Predictive Control architectures yielding optimal accuracy (+5.8%). The underperformance of adaptive controllers (−4.2%) suggests intrinsic limitations in reconciling adaptation mechanisms with the thermomechanical hysteresis characteristics of conventional SMA systems.

MSMA systems exhibit pronounced sensitivity to implementation strategy, with sensorless approaches providing substantial performance improvements (+5.0%) compared to sensor-based methodologies (−6.5%). Similarly, pure artificial intelligence implementations demonstrate superior performance (+5.0%), while iterative learning control frameworks show marked deficiencies (−8.9%).

The differential response patterns, particularly in MPC implementations (SMA: +5.8%; MSMA: −7.7%), underscore the material-specific nature of optimal control strategy selection, establishing quantitative benchmarks for application-specific implementation in smart material actuation systems.

### 3.3. Types of Artificial Intelligence

#### 3.3.1. Feedforward Neural Networks

Feedforward Neural Networks (FNNs) represent the fundamental architectural paradigm employed in SMA actuator control applications, characterized by a unidirectional information propagation pathway without feedback mechanisms. These computational structures are designed to predict system states through supervised learning processes, wherein the network parameters are optimized using datasets containing paired input-output relationships.

**Figure 18:**
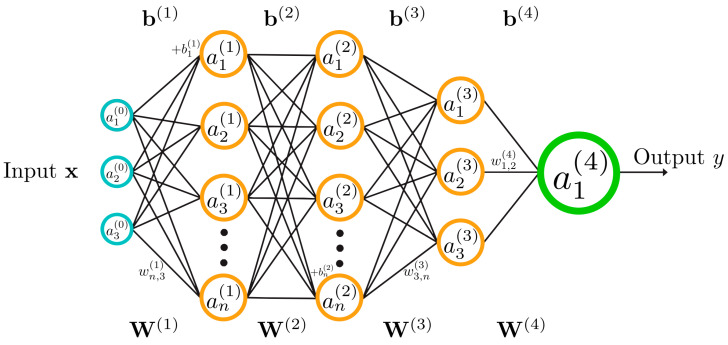
Schematic representation of a feedforward neural network architecture with three hidden layers, illustrating input variables, hidden layer configurations, and output mapping.

The network architecture comprises multiple interconnected layers of artificial neurons [15], with each neuron establishing weighted connections to all neurons in adjacent layers, as schematized in Figure 18. The fundamental computational operation performed by each neuron can be mathematically expressed as follows:

(6) $$y_{j}=\phi \left(\sum \theta_{ij}x_{i}\right)$$

where $y_{j}$ denotes the output of the j-th neuron, $x_{i}$ represents inputs from the preceding layer, ϕ is the non-linear activation function, and $\theta_{ij}$ represents the connection weight parameters between neurons i and j.

The network structure comprises an input layer with neurons corresponding to the number of input variables, one or more hidden layers facilitating complex feature extraction, and an output layer proportional to the desired output dimensions. The hidden layers enable the generation of sophisticated non-linear combinations of input variables, facilitating the approximation of complex functions. The network’s capacity for function approximation is governed by critical hyperparameters including the number of layers, neurons per layer, and activation function specifications.

Common activation functions implemented in SMA control applications include the sigmoid function (logsig):

(7) $$\phi \left(x\right)=\frac{1}{1+e^{-x}}$$

which maps inputs to outputs in the range [0, 1], and the hyperbolic tangent function (tansig):

(8) $$\phi \left(x\right)=\tanh\left(x\right)$$

mapping inputs to the range [−1, 1]. The latter is more frequently utilized due to its implementation in computational environments such as MATLAB R2010b and later versions [58]. For specialized applications, radial basis activation functions [48] have demonstrated efficacy, particularly the Gaussian formulation:

(9) $$\phi \left(x\right)=\exp\left(-\frac{{\left(x-\mu \right)}^{2}}{2\delta^{2}}\right)$$

characterized by center (μ) and width (δ) parameters that provide enhanced flexibility in modeling complex non-linearities prevalent in SMA behavior.

While FNNs demonstrate robust capabilities in approximating non-linear relationships, they exhibit inherent limitations in modeling time-dependent phenomena such as hysteresis, where identical inputs may yield different outputs contingent upon the system’s historical states [34]. This limitation has been addressed through supplementary approaches, including the incorporation of directional “tag” inputs indicating phase transformation direction [36], or implementation of more advanced recurrent neural network architectures [54] that explicitly account for temporal dependencies in SMA behavior.

Training is a fundamental process whereby the weights of a neural network are modified to connect a set of input and output data. Neural network training for SMA control applications employs supervised learning approaches using input–output data pairs. This process may be implemented through the following:

1. **Offline Training**: Model parameters are established using historical data before deployment, with limited adaptation capability during operation.
2. **Online Training**: Continuous parameter updates based on real-time operational data, enabling adaptation to changing system dynamics.

For FFNs, training relies on optimization methods that operate on a cost function, such as mean square error (MSE). For multi-layer networks, the training algorithms require the use of backpropagation (BP), which propagates the error from the output layers to the previous ones, allowing the weights to be updated accordingly.

The main algorithms are as follows:

1. **Gradient descent method**: an iterative method that updates the weights in the opposite direction to that of the cost function gradient [55]. The most common cost function is the MSE. Its speed is regulated by the learning rate, but high values can cause overshooting. It is often modified for online training. The gradient descent algorithm can be modified to be used online, by iteratively applying the training method to subsequent batches of data after the initial training [48].
2. **Levenberg–Marquadt (LM):** the most widely used algorithm. It is a combination of the gradient descent method with the Newton–Raphson method, an alternative method which considers curvature of the cost function through its Hessian matrix to update the weights. The LM algorithm combines the robustness of gradient descent with the convergence speed of Newton–Raphson [35].

#### 3.3.2. Recurrent Neural Networks

Recurrent Neural Networks (RNNs) represent a class of neural networks specifically designed for sequential data processing. Unlike feedforward networks, RNNs are characterized by feedback connections that enable “memory” of past inputs and outputs, making them suitable for modeling time-evolving phenomena such as hysteresis, where identical inputs can yield different outputs depending on the system’s history.

Among various architectures, Non-linear Autoregressive with Exogenous inputs (NARX) and Non-linear Autoregressive Moving Average with Exogenous inputs (NARMAX) models are prominent. In a NARX model, the current system output is modeled as a non-linear function of past values:

(10) $$y\left(k\right)=f\left[u\left(k-1\right),\ldots ,u\left(k-l\right),y\left(k-1\right),\ldots ,y\left(k-m\right)\right]$$

**Figure 19:**
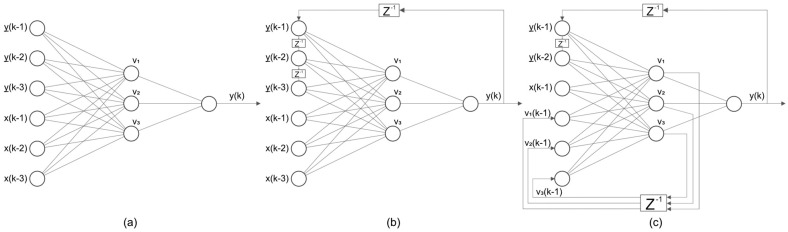
Structures used for NARX networks, [54]: (**a**) serial Jordan network, (**b**) parallel Jordan network, (**c**) Jordan–Elman network.

The network features a single output neuron and l + m input neurons, where l and m represent the memory depth for inputs and outputs. Several variants exist:

1. Jordan Network: Feedback from the output layer, in either serial (using actual outputs, Figure 19a) or parallel (using network-generated outputs, Figure 19b) configurations;
2. Elman Network: Feedback from the hidden layer;
3. Jordan–Elman Network: Feedback from both hidden and output layers, Figure 19c.

Recurrent networks face limitations in time horizon memory due to vanishing or exploding gradient problems.

The NARMAX model extends NARX by incorporating moving averages of inputs. Modeling error from external sensors can serve as a feedback signal [47], improving response to variations in hysteretic characteristics.

Some implementations utilize wavelet activation functions [39]. A variation of the Elman architecture expands input and feedback signals in Fourier series [30] is as follows:

(11) $$\begin{array}{c} y_{k+1}=w_{0}+w_{1}f_{k}+\overset{n}{\underset{l=1}{\sum }}w_{2l}\sin\left(\pi lf_{k}\right)+\overset{n}{\underset{l=1}{\sum }}w_{2l+1}\cos\left(\pi lf_{k}\right)\\ +h_{0}+h_{1}y_{k}+\overset{m}{\underset{l=1}{\sum }}h_{2l}\sin\left(\pi ly_{k}\right)+\overset{m}{\underset{l=1}{\sum }}h_{2l+1}\cos\left(\pi ly_{k}\right) \end{array}$$

where $w_{l}$ and $h_{l}$ are input and feedback weights, respectively. The network input $f_{k}$ is supplied by a hysteresis operator providing unique values for each point. Recurrent networks can also incorporate operations like integration to capture system dynamics [40].

RNNs require specialized training algorithms. For serial Jordan networks, the use of the LM algorithm is cited [52]. A more complex, multi-step process is indicated for the parallel configuration, employing first the training of an equivalent feedforward network with only input memory elements, then the network is modified into a serial Jordan network by introducing real output memory elements, and it is then retrained, and finally the network’s estimated output is connected to form a parallel Jordan network.

An alternative method is Particle swarm Optimization (PSO), a population-based optimization algorithm [28]. In PSO, a swarm of “particles” (weight configurations) is updated based on the best previous local position and the best current global position in the swarm. PSO can be combined with gradient descent to provide adaptive characteristics to the neural network during its use.

The Hybrid Differential Evolution (HDE) method [42] is also cited: applied for online training, HDE uses a differential evolution (DE) algorithm to find a more accurate initial set of weights, to then refine by using a BP method. The HDE method provides faster convergence rates and lower errors. It can also be used online, by employing BP on newer sets of acquired data.

#### 3.3.3. LSTM (Long Short-Term Memory) Networks

**Figure 20:**
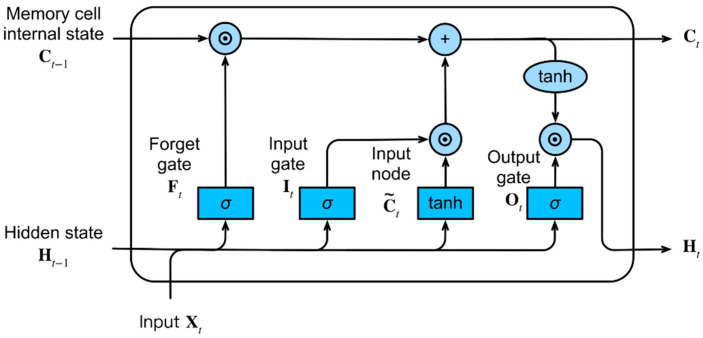
Structure of an LSTM cell showing the three gates and memory flow mechanisms.

LSTM networks overcome the vanishing or exploding gradient problems by specifically designing architecture to learn long-term dependencies in sequential data. The internal architecture differs significantly from conventional neural networks: the neuron is replaced by the LSTM cell (Figure 20), characterized by three “gates” that control operations on long-term and short-term memory signals [57].

The initial operation involves combining outputs from the previous layer’s neurons with the short-term memory from the previous time step. This result is then modified through three distinct gate operations:

1. **Forget Gate:** A sigmoid activation function is applied to the previous operation’s result, yielding a value between 0 and 1. This output is multiplied with the long-term memory from the previous timestep. The purpose of this gate is to modify long-term memory, determining which values should be “remembered.”
2. **Input Gate:** The combination of short-term memory and outputs from the previous layer serves as input to two activation functions sigmoid (producing values between 0 and 1) and hyperbolic tangent (producing values between −1 and 1). The results are multiplied, and this product is added to the long-term memory modified by the forget gate, producing an updated long-term memory value.
3. **Output Gate:** The updated long-term memory undergoes modification through a hyperbolic tangent activation function, while the short-term signal is modified through a sigmoid activation function. These outputs are multiplied to produce a modified short-term memory signal.

This architecture ensures that relevant information can be maintained over extended periods, thus overcoming limitations typical of classical recurrent neural networks. Within the cell, each connection can incorporate weights and biases, making the cell’s behavior fully customizable for various usage conditions.

The fundamental characteristics of LSTM networks can be integrated with other neural network types. For instance, the recursive modeling capabilities of NARX or NARMAX networks can be combined with the LSTM logical structure, creating networks particularly suited for modeling systems with complex temporal behaviors [51]. This approach has been effectively applied to MSMA actuator control applications.

For LSTM networks, training is reported to use gradient descent algorithms [51]. A modification of the gradient descent algorithm adds an inertia term, providing greater robustness.

#### 3.3.4. Reinforcement Learning

Reinforcement Learning (RL) represents a distinct artificial intelligence paradigm characterized by unsupervised training. It operates through an interaction cycle where an agent performs actions in an environment and, by observing consequences in the form of states and rewards, adapts its behavioral strategy [59]. The primary objective is to learn a policy—a strategy allowing the agent to determine actions that maximize expected long-term reward. The RL process involves an agent perceiving the current environment state and selecting an action based on its policy. The environment responds by evolving to a new state and providing a reward signal. The agent uses this sequence to update its policy, aiming to maximize cumulative reward. A central concept is the value function $Q\left(s,a\right)$, which estimates the expected value of taking a specific action in a given state. Another fundamental element is the trade-off between exploration (trying new actions) and exploitation (using known high-reward actions).

**Figure 21:**
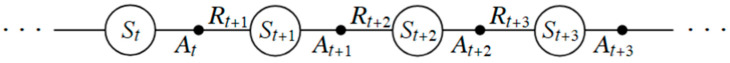
Transition sequence for the SARSA method showing state-action-reward-state-action flow [59].

One algorithm used for SMA actuator control is SARSA (State-Action-Reward-State-Action) [41]. This on-policy algorithm learns the value of the policy it is currently following. Learning occurs through experience (Figure 21), in the form of transition sequences: at each step, the agent is in state $S_{t}$, selects action $A_{t}$ based on the current policy, receives reward $R_{t+1}$, and transitions to new state $S_{t+1}$, where it selects action $A_{t+1}$. Actions are chosen using an ϵ−greedy policy, where with probability 1−ϵ, the agent selects the action with the currently highest reward value (exploitation), while with probability $\epsilon \in \left[0;1\right]$ the agent selects a random action (exploration).

At each step, the policy is updated through reward adjustments: for each state–action pair, with S’ and A’ representing future states and actions:

(12) $$\Delta Q\left(S,A\right)=\alpha \left[R+\gamma Q\left(S^{\prime },A^{\prime }\right)-Q\left(S,A\right)\right]$$

Following the update, the process advances to the next step, with $S_{t}$ equal to $S_{t+1}$ from the previous step and $A_{t}$ equal to $A_{t+1}$ from the previous step. The policy update rule contains two parameters: α, the learning rate determining how quickly $Q\left(S,A\right)$ values are updated, and γ, the discount factor determining the importance of future rewards relative to immediate ones.

A strength of the SARSA algorithm is its on-policy nature, where the policy is modified based on the results of its use. SARSA depends directly on interaction with the environment and does not require an explicit model of environmental dynamics. The fundamental structure of RL algorithms, characterized by an agent responding to environmental stimuli without knowing environment dynamics, is well-suited for formulating innovative feedback controllers that evolve their operating mechanisms with use, thus exhibiting adaptability [41].

The SARSA algorithm has limitations: its on-policy nature can be a weakness since the learned policy is directly influenced by the exploration policy, potentially making convergence slower compared to off-policy methods. Additionally, if the exploration policy is not sufficiently diverse, SARSA might converge with a suboptimal policy. SARSA also requires an algorithm to discretize the continuous physical environment into a discrete set of states, such as the k-Nearest Neighbor algorithm [41], where a function approximation is provided by the k values closest to each state, with each value referring to an “attribute”, a degree of freedom necessary for state representation.

#### 3.3.5. Artificial Intelligence Model Performance Analysis

Artificial intelligence (AI) approaches represent a significant advancement in addressing the complex control challenges associated with Shape Memory Alloy (SMA) and Magnetic Shape Memory Alloy (MSMA) actuators. These methodologies have demonstrated particular efficacy in modeling and compensating for the non-linear behavior and hysteresis effects inherent to smart material actuation systems.

AI methodologies have been implemented across diverse functional domains within SMA control architectures, including the following:

1. **Material Behavior Modeling and Hysteresis Compensation** [37]: Neural networks provide accurate mathematical representations of complex thermomechanical coupling phenomena.
2. **Sensorless Control Implementation** [38,46]: AI enables position estimation based on secondary signals such as electrical resistance or generated force, eliminating the need for external position sensors.
3. **Feedforward Control Systems** [55]: Direct neural network control without intermediate conventional control elements.
4. **Closed-Loop Control Through Reinforcement Learning** [41]: Adaptation through environmental interaction rather than explicit mathematical modeling.
5. **Enhanced Model Predictive Control (MPC)** [30] and **Iterative Learning Control (ILC)** [39]: Integration of neural networks into advanced control frameworks for improved performance.

Additionally, adaptive controller characteristics can be achieved through online training techniques that continuously update system parameters based on real-time operational data [49].

Neural networks constitute the predominant AI approach for SMA control, comprising approximately 96% of implementations reported in the literature. These computational models, inspired by biological neural structures, demonstrate exceptional capability in approximating complex non-linear functions with multiple variables [37].

The fundamental architecture consists of interconnected artificial neurons organized in layers. Each neuron aggregates weighted signals from preceding layers and processes this input through a non-linear activation function, introducing the necessary non-linearity for modeling complex systems. Learning occurs through iterative weight adjustment to minimize a cost function representing the difference between desired and actual outputs [49].

Neural network implementations for SMA control have evolved across three primary architectural paradigms:

1. **Feedforward Neural Networks**: The earliest and most prevalent architecture, characterized by unidirectional information flow from input to output layers.
2. **Recurrent Neural Networks**: Enhanced architectures incorporating feedback connections to capture temporal dependencies crucial for modeling hysteresis effects.
3. **Long Short-Term Memory (LSTM) Networks**: Advanced recurrent architectures with specialized memory cells designed to model long-term dependencies in sequential data.

The selection of network architecture and hyperparameters is highly application-specific, necessitating systematic validation processes such as K-Fold Cross Validation [33] or custom algorithms for recurrent networks [54] to prevent underfitting or overfitting.

The distribution between online training and offline training approaches shows approximately 54% offline and 46% online implementations in the literature. The predominant training algorithm is Levenberg–Marquardt (42%), followed by gradient descent methods (25%) and custom algorithms (21%).

The comprehensive classification of AI methodologies for SMA and MSMA actuator control presented in Table 5 provides a systematic framework for evaluating implementation characteristics, training approaches, and performance outcomes across literature.

**Figure 22:**
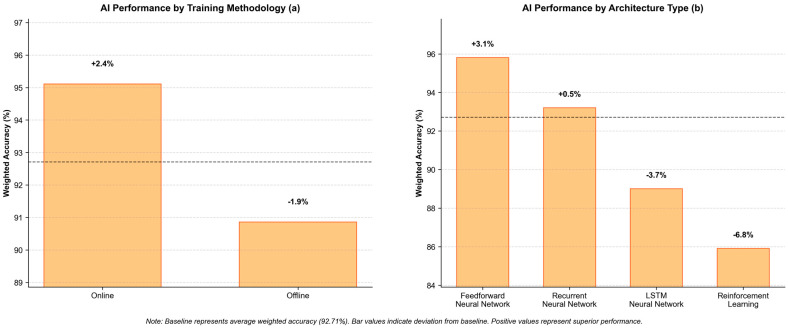
Quantitative comparison of AI methodologies for SMA/MSMA actuator control, where dashed line represents the average Weighted Accuracy (%): (**a**) online vs. offline training performance and (**b**) performance across architectural paradigms (feedforward, recurrent, LSTM, RL).

Performance analysis across AI implementations reveals a mean weighted accuracy of 92.71%, with online training algorithms demonstrating superior performance compared to offline approaches. Figure 22 presents a quantitative comparison of AI methodologies for SMA/MSMA actuator control. Panel (a) demonstrates that online training approaches yield superior performance (+2.40%) compared to offline methodologies (−1.85%), likely due to their continuous adaptation capabilities. Panel (b) reveals significant performance differentials across architectural paradigms, with feedforward networks exhibiting optimal accuracy (+3.10%), while LSTM implementations (−3.70%) and reinforcement learning approaches (−6.80%) demonstrate substantial limitations relative to the 92.71% baseline accuracy. These findings suggest that traditional neural network architectures with online training provide the most effective control framework for smart material actuator systems.

**Figure 23:**
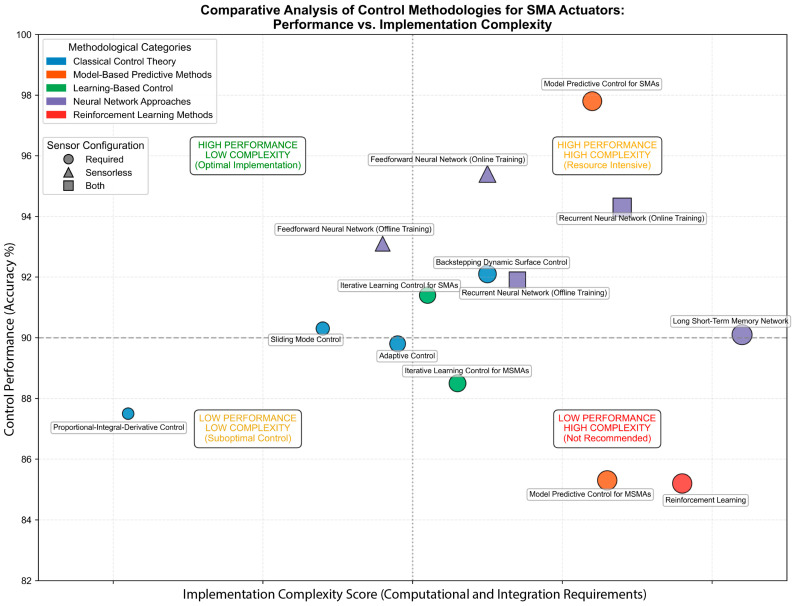
Comparative analysis of control methodologies for SMA actuators illustrating the relationship between control performance and implementation complexity.

Figure 23 presents a systematic performance against complexity mapping of control methodologies for Shape Memory Alloy (SMA) actuators. This analytical framework reveals distinct methodological clustering across performance thresholds and implementation complexity boundaries.

The visualization demonstrates that Model Predictive Control (MPC) approaches for conventional SMAs occupy the high-performance/high-complexity quadrant, exhibiting superior accuracy despite substantial computational requirements. Conversely, their MSMA counterparts demonstrate diminished performance metrics, underscoring the material-specific nature of control optimization challenges in magneto-mechanical systems. Neural network implementations—particularly Feedforward and Recurrent architectures with online training protocols—achieve exceptional performance at moderate complexity levels. These sensorless implementations leverage intrinsic material properties for state estimation, eliminating external sensor dependencies. Classical control methodologies, while generally exhibiting lower implementation complexity, demonstrate variable performance characteristics that frequently fall below established performance thresholds. This analytical framework provides critical implementation insights for resource-constrained applications where computational efficiency must be balanced against performance requirements. The identified optimal methodologies establish a foundation for application-specific optimization in rehabilitation robotics, aerospace systems, and other domains where SMA actuation offers compelling advantages over conventional approaches.

#### 3.3.6. AI Control Limitations and Future Research Roadmap in SMA Actuated Systems

While artificial intelligence methodologies have demonstrated considerable efficacy in SMA actuator control applications, several fundamental limitations constrain their practical implementation across both conventional and magnetic SMA systems:

- **Computational Resource Requirements:** Real-time AI control, particularly online learning approaches, demands significant computational resources that may exceed the capabilities of embedded control systems. This limitation becomes critical in applications requiring rapid response times or operating under strict power consumption constraints, regardless of the specific SMA actuation mechanism employed.
- **Training Data Quality and Comprehensiveness:** Neural network-based controllers require extensive, high-quality training datasets that capture the full operational envelope of SMA behavior. Experimental generation of such datasets is time-intensive and resource-demanding, often requiring sophisticated testing apparatus and precise environmental control. Poor training data quality directly compromises controller performance across all SMA system variants.
- **Black-Box Behavior and Interpretability:** AI controllers, particularly deep neural networks, operate as black-box systems where the relationship between inputs and outputs lacks physical interpretability. This opacity creates challenges for system validation, fault diagnosis, and safety certification in critical applications where understanding the control logic is essential.
- **Hysteresis Model Limitations:** While AI approaches demonstrate superior hysteresis compensation compared to linear methods, they struggle with complex, multi-loop hysteresis characteristics that can emerge under specific loading conditions or after extended cycling. The path-dependent nature of severe hysteresis can exceed the modeling capabilities of current neural network architectures.
- **Real-Time Adaptation Constraints:** Online learning algorithms face the exploration–exploitation trade-off, where the controller must balance learning new behaviors against maintaining stable performance. In safety-critical applications, excessive exploration can lead to system instability or performance degradation.
- **Model Degradation Over Time:** SMA materials exhibit property evolution due to thermal cycling, mechanical fatigue, and microstructural changes. AI controllers trained on initial material properties may gradually lose accuracy as material characteristics evolve, requiring periodic retraining or adaptive mechanisms that current implementations inadequately address.
- **Integration with Physical Constraints:** Many AI approaches lack explicit incorporation of fundamental physical limits such as maximum transformation strain, critical stress thresholds, or thermal damage temperatures. This can result in control commands that, while mathematically optimal, are physically unrealistic or potentially damaging to the actuator system.

While artificial intelligence methodologies have demonstrated considerable efficacy in SMA actuator control applications, several fundamental limitations constrain their practical implementation. Based on our systematic analysis of these limitations, the following research roadmap emerges:

**Immediate Research Priorities (1–3 years):**

- Development of standardized benchmarking datasets incorporating diverse SMA compositions, hysteresis characteristics, and operational conditions for systematic AI algorithm comparison;
- Investigation of lightweight neural architectures achieving <2% RMSE while reducing computational requirements by 80% for embedded microcontroller deployment;
- Systematic comparison of physics-informed neural networks vs. purely data-driven approaches to establish optimal integration strategies for SMA control;
- Creation of unified performance metrics combining tracking accuracy, energy efficiency, and robustness for meaningful cross-study comparisons;
- Development of adaptive fatigue compensation algorithms addressing the current gap in material degradation management over extended cycling.

**Medium-term Research Goals (3–7 years):**

- Integration of multi-physics thermomechanical modeling with AI control architectures for improved performance prediction and compensation;
- Development of material-specific neural architecture search algorithms addressing the observed performance differentials between SMA (+5.8%) and MSMA (−7.7%) systems;
- Exploration of federated learning approaches enabling cross-system knowledge transfer between different SMA actuator configurations and applications;
- Implementation of multi-modal sensor fusion frameworks combining electrical resistance, temperature, force, and vision-based measurements for enhanced state estimation;
- Creation of safe online learning algorithms with formal stability guarantees addressing the current limitation where only 46% of implementations utilize real-time adaptation.

**Long-term Research Vision (7+ years):**

- Autonomous SMA system design using AI-driven optimization for actuator geometry, material selection, and control architecture co-design;
- Integration of SMA control with broader smart material ecosystems enabling coordinated multi-actuator systems for complex applications;
- Development of explainable AI approaches providing real-time control decision insights for safety-critical aerospace and biomedical applications;
- Creation of digital twin frameworks incorporating material evolution models for predictive maintenance and performance optimization;
- Establishment of industry-standard AI control platforms enabling plug-and-play SMA actuator integration across diverse engineering applications.

## 4. Conclusions

This paper has presented a systematic review and performance analysis of artificial intelligence control methodologies applied to Shape Memory Alloy (SMA) actuators. By integrating data from a range of studies spanning nearly two decades, the review has elucidated the intricate interplay between the inherent nonlinearities and hysteresis effects of SMAs and the advanced control strategies developed to manage these challenges. The convergence of traditional control theory with modern AI approaches has provided a promising avenue for achieving both high accuracy and adaptive performance in smart actuator systems.

One of the critical insights gained from the comparative analysis is the effectiveness of dynamic closed-loop control architectures over static or open-loop systems. The evaluation framework, which employs normalized Root Mean Square Error (RMSE) metrics along with a weighted accuracy scoring system, clearly demonstrates that AI-based strategies particularly those that blend feedforward neural network models with adaptive online training consistently outperform conventional control methods in managing the thermomechanical complexities of SMA actuators. This reflects a paradigm shift where the integration of real-time learning capabilities compensates for material nonlinearity and hysteresis, leading to improved tracking precision and operational stability. Recent developments in neural network architectures have introduced sophisticated approaches to SMA control that build upon foundational methodologies while addressing specific challenges in smart material actuation. Contemporary LSTM implementations, exemplified by Zhou et al. (2024) [57] and Su et al. (2024) [51], incorporate specialized memory cell configurations optimized for managing the complex temporal dependencies inherent in hysteretic systems. These architectures demonstrate that advanced memory management can achieve comparable performance to traditional approaches while providing enhanced adaptability to system evolution.

Hybrid AI-classical integration represents another contemporary advancement, with recent MPC frameworks incorporating neural network-based non-linear models that address dead-zone compensation and anti-windup mechanisms. These implementations demonstrate how modern AI architectures can enhance classical control approaches rather than replacing them entirely, achieving performance improvements while maintaining the stability guarantees of established control theory.

However, our quantitative analysis reveals that architectural sophistication does not guarantee superior performance in SMA control applications. Contemporary LSTM networks achieve −3.7% performance relative to baseline accuracy, while traditional feedforward architectures maintain +3.1% superior performance compared to more sophisticated recurrent implementations. This finding underscores the importance of application-specific validation rather than assuming that general AI advances translate directly to smart material control benefits.

Despite these advancements, several limitations remain inherent in current AI-controlled SMA systems. While the majority of the reviewed studies successfully mitigate issues related to position sensing and signal processing, challenges persist in optimizing control algorithms for varying external conditions such as load variations, temperature fluctuations, and mechanical fatigue. The reliance on offline training in a significant number of implementations also highlights the need for more robust online adaptation mechanisms that can continuously update system parameters in response to evolving operational demands.

Looking ahead, future research should focus on the development of hybrid models that combine the strengths of various AI architectures including feedforward, recurrent, and reinforcement learning paradigms to achieve a more comprehensive understanding of the underlying material dynamics. In addition, further exploration into sensorless control approaches and the integration of multi-modal sensing systems may provide substantial benefits by reducing hardware complexity and enhancing the overall efficiency of SMA-based actuation. The application of advanced optimization techniques and robust model predictive control frameworks is also anticipated to play a crucial role in addressing the current performance constraints, thereby paving the way for broader industrial applications.

In conclusion, the fusion of artificial intelligence with SMA actuator control presents both a compelling research opportunity and a significant technological advancement. The findings of this paper underscore the potential of AI to transform the landscape of smart material applications by offering innovative solutions to long-standing challenges in actuator control. Continued interdisciplinary collaboration and the development of integrative methodologies are essential for translating these research insights into scalable, real-world systems that meet the increasingly demanding performance standards in aerospace, biomedical, robotics, and other engineering fields.

## Figures and Tables

**Table 1:** Position sensing technologies for SMA Actuators.

Technology [References]	Measurement Principle	Characteristics	Precision	Applications	Limitations
**Linear Variable Differential Transformer (LVDT)** [36,37,38,39]	Electromagnetic induction with ferromagnetic core positioned within three coils (one primary AC-driven, two secondary connected in opposite phase); core displacement generates proportional voltage differential	Most widely deployed position sensing technology in SMA actuation systems; provides direct measurement of linear displacement	Dependent on output voltage assessment instrumentation	SMA contraction/expansion measurement [36]; MSMA displacement monitoring [39]	Form factor limitations for miniaturized or lightweight applications [38]
**Laser Displacement Sensors** [34,40]	Non-contact measurement evaluating position changes in laser beam reflected from mobile elements (typically reflective discs mounted at actuator endpoints)	Eliminates mechanical coupling with experimental apparatus; commercial implementations include Keyence IL series and IDEC MX1A/B models	Micron-level repeatability with application-specific measurement ranges	Longitudinal displacement measurement without mechanical interference [34,40]	Similar form factor challenges as LVDTs for miniaturized applications
**Linear Potentiometers** [41,42]	Resistive sensing determining probe position through internal electrical resistance variations	Commercial models (e.g., Linear Position Transducer) offer flexible measurement ranges (150–1420 mm)	Variable based on component specification	Rectilinear displacement measurement in larger-scale applications [41]	Lower precision than laser or LVDT alternatives
**Rotary Encoders** [31,43,44]	Patterned disc with transparent/opaque regions modulating light detection during rotation, generating electrical signals converted to angular position	Can provide direct rotational measurement or indirect translational monitoring when coupled with pulleys; can be integrated within robotic systems	Up to 2500 pulses per revolution (e.g., Omron E6B2-CWZ1X)	Direct angular position measurement [43]; indirect translational monitoring via pulleys [44]; robotic system integration [31]	Limited to rotational measurement without conversion mechanisms
**Magnetic Tracking Systems** [29,45]	Electromagnetic field detection for spatial positioning	Enables six-degree-of-freedom tracking through miniaturized sensors (e.g., trakSTAR 3D Magnetic Tracking System)	Sub-millimeter 3D positioning capability	Three-dimensional position assessment for complex actuation systems [29]	Susceptible to electromagnetic interference; requires specialized infrastructure
**High-Speed Camera Systems** [33]	Computer vision analysis with Kinovea kinematic software processing	Emerging methodology enabling non-contact multi-point tracking	Dependent on camera resolution and processing algorithms	Spatial position evaluation of robotic systems; trajectory and velocity characterization [33]	Requires line-of-sight; computationally intensive; lighting-dependent

**Table 2:** Alternative sensing methodologies for SMA actuator systems.

Technology [References]	Measurement Principle	Implementation Methodologies	Advantages	Applications	Limitations
**Electrical Resistance Monitoring (Self-Sensing)** [27,34,38,46]	Exploitation of phase-dependent resistivity differential between martensitic (higher) and austenitic (lower) phases	**Programmable Power Supply**: Controls one electrical parameter while measuring another; applies Ohm’s law for resistance calculation. **Series Resistor**: In constant-voltage scenarios, uses calibrated resistor for indirect current measurement via voltage sensing	Eliminates external position sensor requirements; enables system miniaturization; leverages intrinsic material properties	Phase transformation monitoring [27]; position estimation during actuation [38]; antagonistic wire configuration assessment [34]	Requires continuous energization; extends cooling times; necessitates instrumentation integration with activation signal
**Force Measurement** [34,46]	Direct stress/strain quantification during phase transformation	Load cell integration within actuation train	Enables correlation between material deformation and actuator position; provides structural stress monitoring capabilities	Material deformation correlation [46]; structural stress monitoring [34]	Requires mechanical integration; introduces additional mass/complexity
**Temperature Monitoring** [41,46]	Direct thermal state assessment of SMA elements	Thermocouple attachment to SMA elements	Provides actuation state verification; enables temperature-based control signal generation	Control signal generation [41]; operational state verification [46]	Measurement bias related to attachment methodology; limited spatial resolution
**Airflow Measurement** [46]	Quantification of convective cooling conditions	Cup anemometer deployment within experimental setup	Enables characterization of cooling system influence on actuator response dynamics	Cooling system performance evaluation [46]	Limited to laboratory environments; primarily for experimental characterization
**Data Acquisition Systems** [28,30,31,33,35,37,41]	Analog-to-digital signal conversion and processing	Commercial systems (National Instruments [28], dSPACE [31], Advantech [30]); embedded approaches (Arduino Nano [33], Jetson Nano [35])	Integrates sensor networks with control systems; enables real-time data processing and control implementation	Experimental characterization [37]; control system implementation [41]; miniaturized implementations [33,35]	System complexity; calibration requirements; platform constraints

**Table 3:** Comparative analysis of conventional control strategies for SMA/MSMA actuators, including PID, SMC, BDSC, Adaptive Control, MPC, and ILC, with classifications, working principles, advantages, limitations, and applicability.

Control Strategy [References]	Classification	Working Principle	Key Advantages	Limitations/Challenges	Applicability to SMA/MSMA
**Proportional-Integral-Derivative (PID)** [37,48]	Linear, feedback-based	Generates control signal based on error between setpoint and actual output; combines proportional response to current error, integral action on accumulated error, and derivative action based on rate of change	Established industrial standard; intuitive parameter tuning; responsive to transient disturbances; well-documented implementation protocols	Performance degradation in non-linear regimes; steady-state error persistence without integral component; susceptibility to derivative noise amplification; integral windup during saturation conditions	Limited efficacy for SMA/MSMA systems due to pronounced hysteresis and non-linear thermomechanical coupling; requires supplementary compensation mechanisms
**Sliding Mode Control (SMC)** [46,49]	Non-linear, robust	Constrains system state trajectory onto a predefined sliding manifold through discontinuous control actions; operates in sequential reaching and maintenance phases	Inherent robustness against parametric uncertainties and external disturbances; reduced-order equivalent control problem; rapid convergence characteristics; invariance to matched uncertainties	High-frequency oscillations (chattering) at control discontinuities; potential for actuator degradation due to rapid switching; requires accurate sliding surface design; sensitive to unmodeled dynamics	Highly compatible with SMA/MSMA actuators due to robust performance under thermomechanical uncertainties and phase transformation non-linearities
**Backstepping Dynamic Surface Control (BDSC)** [50]	Non-linear, recursive	Implements systematic stabilization through recursive Lyapunov-based design; decomposes complex non-linear systems into cascaded subsystems with virtual control variables; employs low-pass filtering for derivative estimation	Superior tracking precision; systematic design methodology for complex non-linear systems; analytical stability guarantees; robustness against bounded disturbances; reduced computational burden compared to standard backstepping	Requires comprehensive analytical system model; increased design complexity with system order; potential performance degradation under severe model uncertainties; non-trivial implementation requirements	Exceptionally suitable for SMA/MSMA actuators due to systematic handling of non-linearities and hysteresis effects; accommodates complex thermomechanical coupling phenomena
**Adaptive Control** [29,43]	Parameter-adjusting, dynamic	Modifies controller parameters systematically according to performance metrics and estimation algorithms; utilizes reference models (MRAC) or direct adaptation mechanisms; incorporates parameter convergence safeguards	Autonomously accommodates parametric variations and uncertainties; minimal a priori system knowledge requirements; performance preservation under time-varying conditions; resilience to gradual system degradation	Parameter convergence not universally guaranteed; potential instability during rapid adaptation; susceptibility to disturbance-induced parameter drift; complex stability analysis requirements	Highly effective for SMA/MSMA systems exhibiting parameter variations due to thermal cycling, fatigue, or environmental conditions; compensates for time-variant hysteresis characteristics
**Model Predictive Control (MPC)** [30,51,52]	Optimization-based, predictive	Formulates control as a receding horizon optimization problem; predicts future system behavior using dynamic models; minimizes cost functions subject to explicit constraints; implements only first element of optimal control sequence	Explicit constraint handling capability; multivariable system accommodation; anticipatory disturbance compensation; systematic performance-constraint trade-off management; optimality guarantees within prediction horizon	Substantial computational requirements for online optimization; performance critically dependent on model fidelity; implementation complexity increases with horizon length and constraint complexity; potential suboptimality under model-plant mismatch	Well-suited for SMA/MSMA applications requiring constraint enforcement (thermal, mechanical, electrical) while maintaining performance objectives; particularly effective when augmented with accurate thermomechanical models
**Iterative Learning Control (ILC)** [39,47,53]	Memory-based, repetitive	Exploits task repetition to progressively refine control signals; utilizes error information from previous iterations to improve subsequent performance; implements memory-based feedforward corrections derived from historical execution data	Progressive performance enhancement for repetitive tasks; minimal model dependence; effective compensation for repeatable disturbances; potential for perfect tracking under ideal conditions	Strictly limited to repetitive operations with consistent initial conditions; performance degradation under non-repetitive disturbances; potential error amplification without appropriate filtering; memory-intensive implementation	Particularly advantageous for cyclic SMA/MSMA applications with repetitive actuation requirements; effective for compensating repeatable hysteresis effects; suitable for precision positioning tasks

**Table 4:** Performance metrics and methodological classifications of SMA/MSMA control strategies, including tracking performance (RMSE%), sensor utilization, feedback mechanisms, and accuracy scores.

Author and Year [Reference]	Title	Position Sensor Utilization	Feedback	Static/Dynamic Methodology	Control Algorithm	Tracking Performance (RMSE%)
**Song, G. et al., 2003** [28]	A Neural Network Inverse Model for a Shape Memory Alloy Wire Actuator	Sensorless	No	Static	AI or AI combination	Sinusoidal signal: 7%. Accuracy score: 0.93
**Song, G. et al., 2003** [36]	Precision tracking control of shape memory alloy actuators using neural networks and a sliding-mode based robust controller	With position sensor	Yes	Static	Non-linear controllers (SMC) and AI	Sinusoidal signals: 5.1%. Evaluation conducted with various amplitudes and frequencies. Accuracy score: 2.87
**Ma, N. et al., 2004** [27]	Position control of shape memory alloy actuators with internal electrical resistance feedback using neural networks	Sensorless	Yes	Static	Linear controllers (PD) and AI	Multistep signal: 7%. Accuracy score: 1.86
**Asua, E. et al., 2008** [37]	Neural network-based micropositioning control of smart shape memory alloy actuators	With position sensor	Yes	Static	Linear controllers (PI) and AI	Square wave and ramp signals: <1%. Accuracy score: 1.98
**Asua, E. et al., 2010** [38]	Sensorless Control of SMA-based Actuators Using Neural Networks	Sensorless	Yes	Static	Linear controllers (PID) and AI	Ramp and step signals: <6%. Accuracy score: 2.82
**Kirkpatrick, K. et al., 2011** [41]	Active Length Control of Shape Memory Alloy Wires Using Reinforcement Learning	With position sensor	Yes	Dynamic	AI or AI combination	Multistep signals: <18%. Accuracy score: 1.64
**Tai, N. et al., 2012** [30]	A hysteresis functional link artificial neural network for identification and model predictive control of SMA actuator	With position sensor	Yes	Dynamic	MPC and AI algorithms	Multistep and sinusoidal signals: <0.2%. Accuracy score: 2.99
**Mai, H. et al., 2013** [40]	Adaptive online inverse control of a shape memory alloy wire actuator using a dynamic neural network	With position sensor	Yes	Dynamic	Linear controllers (P) and AI	Sinusoidal signals: <1%. Evaluation conducted with various amplitudes and frequencies. Accuracy score: 1.98
**Wang, H. et al., 2014** [54]	Innovative NARX recurrent neural network model for ultra-thin shape memory alloy wire	With position sensor	Yes	Static	AI or AI combination	Sinusoidal signals: 3.5%. Evaluation conducted with various amplitudes. Accuracy score: 1.93
**Senthilkumar, P. et al., 2014** [46]	Use of load generated by a shape memory alloy for its position control with a neural network estimator	Sensorless	Yes	Static	Linear controllers (PI with gain scheduling) and AI	Multistep signal: <1.5%. Evaluation conducted with various signal types. Accuracy score: 1.97
**Son, N.N. et al., 2015** [44]	Adaptive displacement online control of shape memory alloys actuator based on neural networks and hybrid differential evolution algorithm	With position sensor	Yes	Dynamic	Linear controllers (PID) and AI	Sawtooth and trapezoidal signals: <7%. Evaluation conducted with various signal types. Accuracy score: 1.86
**Malinga, B. et al., 2015** [29]	ℒ1 adaptive control of a shape memory alloy actuated flexible beam	With position sensor	Yes	Dynamic	Adaptive controllers and AI	Sawtooth or sinusoidal signals: <25%. Evaluation conducted with various signal types. Accuracy score: 1.5
**Narayanan, P. et al., 2016** [31]	Control of a shape memory alloy–actuated rotary manipulator using an artificial neural network–based self-sensing technique	Sensorless	Yes	Static	Linear controllers (PI with gain scheduling) and AI	Complex signal: 7%. Evaluation conducted with various signal types, with variable amplitude and frequency. Accuracy score: 3.72
**Zhou, M. et al., 2017** [55]	Feed-forward control for magnetic shape memory alloy actuators based on the radial basis function neural network model	Sensorless	No	Static	AI or AI combination	Complex signals: <1%. Evaluation conducted with various signal types, with variable amplitude and frequency. Accuracy score: 3.96
**Pan, Y. et al., 2017** [43]	Output-Feedback Adaptive Neural Control of a Compliant Differential SMA Actuator	With position sensor	Yes	Dynamic	Linear controllers (PD), adaptive and AI	Sinusoidal and complex signals: <2%. Evaluation conducted with various signal types, with variable amplitude and frequency. Accuracy score: 3.92
**Bhargaw, H.N. et al., 2021** [34]	Differential resistance based self-sensing recurrent neural network model for position estimation and control of antagonistic shape memory alloy actuator	Sensorless	Yes	Static	Linear controllers (PI) and AI	Multistep and sinusoidal signals: <5%. Evaluation conducted with various signal types, with variable amplitude and frequency. Accuracy score: 3.8
**Ning, K. et al., 2022** [33]	Using inverse learning for controlling bionic robotic fish with SMA actuators	Sensorless	No	Static	AI or AI combination	Not applicable. Experimental error evaluated on the achieved movement velocity <10%.
**Yu, Y. et al., 2022** [56]	Neural-Network-Based Iterative Learning Control for Hysteresis in a Magnetic Shape Memory Alloy Actuator	With position sensor	Yes	Dynamic	Iterative Learning Control and AI	Sawtooth signals: <14%. Evaluation conducted with various amplitudes. Accuracy score: 1.72
**Yu, Y. et al., 2023** [47]	Neural Network Adaptive Control of Magnetic Shape Memory Alloy Actuator With Time Delay Based on Composite NARMAX Model	With position sensor	Yes	Dynamic	Adaptive controllers and AI algorithms	Sawtooth and complex signals: <4%. Evaluation conducted with various signal types, with variable amplitude and frequency. Accuracy score: 3.84
**Yao, M. et al., 2023** [50]	Backstepping Dynamic Surface Control of an SMA Actuator Based on Adaptive Neural Network	With position sensor	Yes	Dynamic	Backstepping controllers and AI	Sinusoidal signal: 5%. Comparison performed on system model. Accuracy score: 0.95
**Yu, Y. et al., 2023** [39]	Neural network based iterative learning control for magnetic shape memory alloy actuator with iteration-dependent uncertainties	With position sensor	Yes	Dynamic	Iterative Learning Control and AI	Complex signals: <15%. Evaluation conducted with various signal types, with variable amplitude and frequency, and variation in load level. Accuracy score: 4.25
**Khan, A.M. et al., 2024** [48]	Adaptive neural network controller for the rotating SMA actuator	With position sensor	Yes	Dynamic	AI or AI combination	Sinusoidal signals: <2%. Evaluation conducted with input and output disturbances. Accuracy score: 1.96
**Zhou, M. et al., 2024** [57]	Neural Network Based Iterative Learning Control for Dynamic Hysteresis and Uncertainties in Magnetic Shape Memory Alloy Actuator	With position sensor	Yes	Dynamic	Iterative Learning Control and AI	Complex signals: <13%. Evaluation conducted with various signal types, with variable amplitude and frequency. Accuracy score: 3.48
**Su, L. et al., 2025** [51]	Neural network-based non-linear model predictive control with anti-dead-zone function for magnetic shape memory alloy actuator	With position sensor	Yes	Dynamic	MPC and AI algorithms	Sinusoidal and sawtooth signals: <13%. Evaluation conducted with various signal types, with variable amplitude and frequency, and variation in load level. Accuracy score: 4.35

**Table 5:** Comprehensive classification of AI methodologies for SMA/MSMA actuator control, including training approaches, AI types, training algorithms, and tracking performance (RMSE%).

Author and Year [Reference]	Training Approach	AI Type	Training Algorithm	Tracking Performance (RMSE%)
**Song, G. et al., 2003** [28]	Offline	Feedforward Neural Network	Levenberg–Marquardt	Sinusoidal signal: 7%
**Song, G. et al., 2003** [36]	Offline	Feedforward Neural Network	Levenberg–Marquardt	Sinusoidal signals: 5.1%. Evaluation performed with various amplitudes and frequencies.
**Ma, N. et al., 2004** [27]	Offline	Feedforward Neural Network	Levenberg–Marquardt	Multistep signal: 7%
**Asua, E. et al., 2008** [37]	Offline	Feedforward Neural Network	Levenberg–Marquardt	Square wave and ramp signals: <1%
**Asua, E. et al., 2010** [38]	Offline	Feedforward Neural Network	Levenberg–Marquardt	Ramp and step signals: <6%
**Kirkpatrick, K. et al., 2011** [41]	Online	Reinforcement Learning	N/A	Multistep signals: <18%
**Tai, N. et al., 2012** [30]	Online	Recurrent Neural Network	Particle Swarm Optimization + Gradient descent	Multistep and sinusoidal signals: <0.2%
**Mai, H. et al., 2013** [40]	Online	Recurrent Neural Network	Custom online algorithm	Sinusoidal signals: <1%. Evaluation performed with various amplitudes and frequencies.
**Wang, H. et al., 2014** [54]	Offline	Recurrent Neural Network	Custom algorithm for recurrent neural network training	Sinusoidal signals: 3.5%. Evaluation performed with various amplitudes.
**Senthilkumar, P. et al., 2014** [46]	Offline	Feedforward Neural Network	Levenberg–Marquardt	Multistep signal: <1.5%
**Son, N.N. et al., 2015** [44]	Online	Recurrent Neural Network	Hybrid differential evolution	Sawtooth and trapezoidal signals: <7%
**Malinga, B. et al., 2015** [29]	Offline	Recurrent Neural Network	Levenberg–Marquardt	Sawtooth or sinusoidal signals: <25%
**Narayanan, P. et al., 2016** [31]	Offline	Feedforward Neural Network	Levenberg–Marquardt	Complex signal: 7%
**Zhou, M. et al., 2017** [55]	Offline	Feedforward Neural Network	Gradient descent	Complex signals: <1%
**Pan, Y. et al., 2017** [43]	Online	Feedforward Neural Network	Custom online algorithm	Sinusoidal and complex signals: <2%. Evaluation performed with various amplitudes and frequencies.
**Bhargaw, H.N. et al., 2021** [34]	Offline	Recurrent Neural Network	Levenberg–Marquardt	Multistep and sinusoidal signals: <5%
**Ning, K. et al., 2022** [33]	Offline	Feedforward Neural Network	Gradient descent	Not applicable. Experimental error evaluated on movement velocity: <10%
**Yu, Y. et al., 2022** [56]	Offline	Recurrent Neural Network	Gradient descent	Sawtooth signals: <14%. Evaluation performed with various amplitudes.
**Yu, Y. et al., 2023** [48]	Online	Recurrent Neural Network	Custom online algorithm	Sawtooth and complex signals: <4%
**Yao, M. et al., 2023** [50]	Online	Feedforward Neural Network	Custom online algorithm	Sinusoidal signal: 5%. Comparison performed on system model.
**Yu, Y. et al., 2023** [39]	Offline	Recurrent Neural Network	Gradient descent	Complex signals: <15%. Evaluation performed with different load levels applied to the actuator.
**Khan, A.M. et al., 2024** [49]	Online	Feedforward Neural Network	Custom online algorithm	Sinusoidal signals: <2%. Evaluation performed with input and output disturbances.
**Zhou, M. et al., 2024** [57]	Online	LSTM Neural Network	Gradient descent	Complex signals: <13%
**Su, L. et al., 2025** [51]	Online	LSTM Neural Network	Gradient descent	Sinusoidal and sawtooth signals: <13%

## Data Availability

The data that support the findings of this study are not publicly available.
